# Immunotherapy with biodegradable nanoparticles encapsulating the oligosaccharide galactose-alpha-1,3-galactose enhance immune tolerance against alpha-gal sensitization in a murine model of alpha-gal syndrome

**DOI:** 10.3389/falgy.2024.1437523

**Published:** 2024-08-09

**Authors:** Michael N. Saunders, Claudia M. Rival, Mahua Mandal, Kayla Cramton, Laila M. Rad, Katarzyna W. Janczak, Laura A. Williams, Amogh R. Angadi, Jessica J. O’Konek, Lonnie D. Shea, Loren D. Erickson

**Affiliations:** ^1^ Department of Biomedical Engineering, University of Michigan, Ann Arbor, MI, United States; ^2^ Medical Scientist Training Program, University of Michigan, Ann Arbor, MI, United States; ^3^ Beirne Carter Center for Immunology Research, University of Virginia, Charlottesville, VA, United States; ^4^ Department of Microbiology, Immunology, and Cancer Biology, University of Virginia, Charlottesville, VA, United States; ^5^ Mary H. Weiser Food Allergy Center, Michigan Medicine, Ann Arbor, MI, United States; ^6^ Department of Chemical Engineering, University of Michigan, Ann Arbor, MI, United States; ^7^ Department of Surgery, University of Michigan, Ann Arbor, MI, United States

**Keywords:** nanoparticle, tolerance, IgE sensitization, carbohydrates, galactose-α-1, 3-galactose, food allergy

## Abstract

IgE antibodies against the mammalian oligosaccharide allergen galactose-α-1,3-galactose (αGal) can result in a severe allergic disease known as alpha-gal syndrome (AGS). This syndrome, acquired by tick bites that cause αGal sensitization, leads to allergic reactions after ingestion of non-primate mammalian meat and mammalian-derived products that contain αGal. Allergen-specific immunotherapies for this tickborne allergic syndrome are understudied, as are the immune mechanisms of allergic desensitization that induce clinical tolerance to αGal. Here, we reveal that prophylactic administration of αGal glycoprotein-containing nanoparticles to mice prior to tick protein-induced αGal IgE sensitization blunts the production of Th2 cytokines IL-4, IL-5, and IL-13 in an αGal-dependent manner. Furthermore, these effects correlated with suppressed production of αGal-specific IgE and hypersensitivity reactions, as measured by reduced basophil activation and histamine release and the systemic release of mast cell protease-1 (MCPT-1). Therapeutic administration of two doses of αGal-containing nanoparticles to mice sensitized to αGal had partial efficacy by reducing the Th2 cytokine production, αGal-specific IgE production, and MCPT-1 release without reducing basophil activation or histamine release. These data identify nanoparticles carrying encapsulated αGal glycoprotein as a potential strategy for augmenting αGal-specific immune tolerance and reveal diverse mechanisms by which αGal nanoparticles modify immune responses for established αGal-specific IgE-mediated allergic reactions.

## Introduction

Alpha-gal syndrome (AGS) is an atypical IgE-mediated food allergy to the oligosaccharide galactose-α-1,3-galactose (αGal) and was first described 15 years ago (1–3). Patients with AGS are mostly adult and are clinically identified due to the presentation of allergic symptoms ranging from urticaria and gastrointestinal discomfort to anaphylaxis starting between 2 and 6 h after ingestion of non-primate mammalian meat, dairy, or other αGal-containing foods (4–7). In the United States, bites from the Lone Star tick, *Amblyomma americanum*, induce IgE sensitization to αGal (8, 9). Importantly, AGS is becoming a global health problem, with increasing cases reported in all continents with additional tick species implicated in αGal sensitization (10). AGS is therefore markedly different from traditional food allergies, which typically arise early in life and involve acute hypersensitivity reactions to protein allergens.

Mammalian meat avoidance is the primary means of AGS clinical management, which can result in significant social and economic consequences (11). A few reports suggest oral immunotherapy with red meat may desensitize AGS patients; however, limited data from these reports did not explore the effects of oral immunotherapy on the modulation of innate and adaptive immune responses associated with IgE-mediated food allergy (12, 13). The identification of the αGal allergen eliciting IgE responses in AGS holds promise for leading to strategies to identify and intervene in individuals at risk with allergen avoidance or other immunomodulatory approaches. Thus, there is an unmet clinical need to determine whether an αGal glycoprotein-containing immunotherapy can effectively desensitize recipients with AGS and identify how immune tolerance involved in this carbohydrate-specific food allergy may differ from food allergies elicited by protein or other carbohydrate allergens.

IgE sensitization to αGal must be studied in mice deficient in α-galactosyltransferase, an enzyme that produces αGal in lower mammals. Thus, α-galactosyltransferase knockout (AGKO) mice are used to study immune mechanisms of αGal IgE sensitization. Tick-induced sensitization to αGal has been demonstrated in AGKO mouse models of tick feeding or injection of tick extracts through the skin (9, 14–16). Previous work by our group demonstrated that AGKO mice immunized with lone star tick extract induced αGal-specific IgE production, which was dependent on cognate CD4^+^ T cell help (14). Furthermore, AGKO mice sensitized to αGal exhibited greater hypersensitivity responses after ingestion of αGal-containing beef extract compared to wild-type mice that do not make IgE antibodies against αGal. Our group and others have demonstrated the ability to intravenously deliver protein cargo to antigen-presenting cells (APCs) in the spleen and liver via biodegradable polymer poly(lactide-*co*-glycolide) (PLG) nanoparticles (NPs) for tolerance induction (17–20). We have shown that prophylactic and therapeutic intravenous delivery of allergen-loaded NPs attenuate allergic responses in murine models of peanut and egg allergy and result in allergen-specific suppression of Th2 cell responses (19, 21). These findings suggest that NPs are a safe and effective immunotherapeutic approach to induce allergen desensitization in protein-based food allergy.

In this report, we tested the effects of NPs containing encapsulated αGal glycoprotein administered prophylactically and therapeutically to desensitize αGal-sensitized AGKO mice. In mice prophylactically administered αGal NPs, suppression of Th2 cytokines IL-4, IL-5, and IL-13 was observed, which correlated with reduced αGal-specific IgE production and hypersensitivity responses, as measured by decreased frequencies and activation of basophils and mast cell reactivity. Therapeutic delivery of αGal NPs to sensitized mice also suppressed Th2 cytokines, αGal-specific IgE production, and mast cell reactivity but did not affect the frequencies of basophils and mast cells. These results demonstrate the ability of αGal NPs to suppress a carbohydrate allergen-specific IgE response when given prophylactically and therapeutically.

## Materials and methods

### Mice

The alpha-1,3-galactosyl transferase^−/−^ (AGKO) mice have been described and were bred on a C57BL/6 background (14, 22, 23). Studies used 2-month-old, age-matched mice of both sexes with a mean weight of 18–22 g; 12–15 mice were randomly allocated to each experimental NP treatment or phosphate-buffered saline (PBS) control group, and 3–5 were naïve mice. No mice were excluded from the analysis. All mice were bred and maintained in the specific-pathogen-free animal facilities at the University of Virginia with the approval of the Institutional Animal Care and Use Committee protocol #3506 and were used in compliance with the Association for Assessment and Accreditation of Laboratory Animals Care policies.

### Generation and characterization of αGal and control nanoparticles

αGal–human serum albumin (αGal–HSA) and control human serum albumin (HSA) NPs were generated as previously described (19). Briefly, a 12.5 w/w% solution of αGal–HSA (αGal-*β*-1,4-GlcNAc-HSA with three atom spacer; Dextra UK) in human serum albumin (Sigma) was prepared and dissolved at 200 mg/ml in PBS for generation of nanoparticles containing the glycoconjugate αGal–human serum albumin (NP-αGal). Alternatively, a 200 mg/mL HSA protein (Sigma) solution in PBS was used as a starting point to produce control HSA NPs. Then, 150 μL of either the αGal glycoprotein or HSA solution was added to 2 ml of 20% w/v 50:50 poly(lactide-co-glycolide)–COOH (i.v. = 0.18; Evonik) dissolved in dichloromethane. Following sonication, 10 mL of 2% w/v poly(ethylene-alt-maleic anhydride) (PEMA; MW 400 kDa; Polysciences, Inc.) was added, and the solution was sonicated again. This solution was subsequently added to a 0.5% w/v PEMA solution and continuously stirred overnight to allow dichloromethane evaporation. The resulting solid αGal or HSA NPs were washed with 0.1 M sodium bicarbonate–sodium carbonate buffer, pH 9.6 (Polysciences, Inc.) and then lyophilized in a cryoprotectant consisting of 3% w/v aqueous D-mannitol and 4% w/v aqueous sucrose. Dynamic light scattering was performed on each batch of NPs using a Zetasizer Nano ZSP to ensure a diameter between 400 and 700 nm, a surface zeta potential of <−35 mV, and a polydispersity index of <0.3.

### Nanoparticle treatment and IgE sensitization

**Figure 1:**
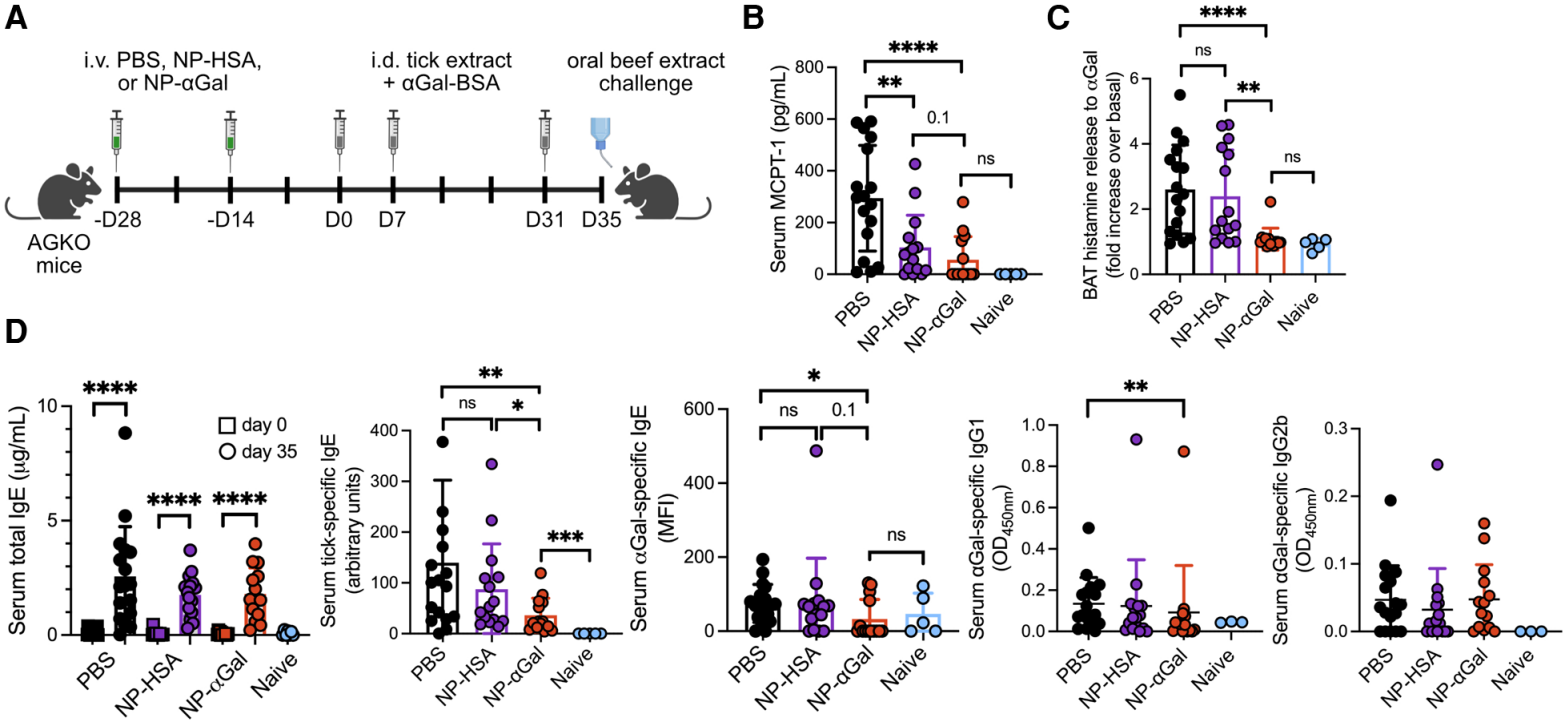
Prophylactic treatment with NP-αGal prevents IgE-mediated allergic responses to αGal. (**A**) Schematic of prophylactic NP treatment in a mouse model of tick extract-induced IgE sensitization to αGal. AGKO mice received two doses of NP-αGal–HSA or control NP-HSA, 2 weeks apart, prior to intradermal injections (i.d.) of whole-body protein extract prepared from lone star seed ticks supplemented with 50 μg αGal-BSA on day 0 and identically prepared booster injections on days 7 and 31. On day 35, mice received an intragastric challenge with beef extract and were analyzed 1.5 h later. (**B**) Concentration of MCPT-1 in the sera of mice after the beef extract challenge measured by ELISA. (**C**) Fold increase in histamine released from the basophil activation test (BAT) with cetuximab measured by ELISA. (**D**) Serum levels of total IgE, tick-specific IgE, and αGal-specific IgE, IgG1, and IgG2b from mice on day 35 measured by ELISA or Luminex. All data are expressed as mean ± SEM. The results shown are representative of two independent experiments. *P* = *0.05, **0.01, and ****0.0001, or ns = not significant, with an unpaired, two-tailed *t*-test.

For prophylactic NP studies, AGKO mice were treated with 2.5 mg of i.v. αGal–HSA NPs or i.v. HSA NPs resuspended in 100 μL of PBS or an equivalent volume (100 μL) of PBS on days −28 and −14 (21) (Figure 1A). Whole-body protein extract was prepared from *A. americanum* seed ticks (Oklahoma Tick Rearing Facility), and the total protein concentration was measured by the BCA assay (Thermo Fisher) as previously described (14). The expression of αGal in tick protein extracts was detected using the 10H8 anti-αGal human IgE mAb (Indoor Biotechnologies) by ELISA; the number of αGal moieties is currently unknown. Thus, each mouse was subsequently given three intradermal (i.d.) injections of 50 μg of total tick extract supplemented with 50 μg of αGal-bovine serum albumin (BSA) on days 0, 7, and 31 to induce IgE sensitization to αGal as previously described (14). On day 35, the mice were administered 250 μg of αGal-containing beef extract (212303; Thermo Fisher) in 100 μL of water via an intragastric (i.g.) challenge, and sera and tissues were subsequently harvested after 90 min for further analysis as previously established (14). For therapeutic NP studies, AGKO mice were given three intradermal (i.d.) injections of 50 μg of whole-body protein extract prepared from *A. americanum* seed ticks and 50 μg of αGal-BSA on days 0, 7, and 31 (Figure 4A). On days 33 and 47, mice were treated with 2.5 mg of i.v. NP-αGal, 2.5 mg of i.v. NP-HSA, or PBS, as described above. On day 63, the mice were given an additional i.d. injection of 50 μg of whole-body protein extract and after 5 days were administered 250 μg of beef extract via an i.g. challenge. Sera and tissues were subsequently harvested after 90 min for further analysis.

### Splenocyte recall assays

Spleens were harvested 90 min after the i.g. beef extract challenge and manually disrupted to generate single-cell suspensions. Red blood cells were depleted using ACK lysing buffer. Lymphocytes were resuspended in culture medium (DMEM, 5% FBS, 2 mM L-glutamine, 1% NEAA, 1 mM sodium pyruvate, 10 mM MOPS, 50 μM 2-mercaptoethanol, 100 IU penicillin, and 100 μg/mL streptomycin) and plated at 800,000 cells per well in tissue culture-treated 96-well flat-bottom plates. Cells were stimulated with 10 μg/mL cetuximab (Lilly) as a source of αGal (4, 24, 25) or left unstimulated in the cell culture medium for use as controls. After 72 h, secretion of cytokines IFN*γ*, IL-4, IL-5, IL-6, IL-10, IL-13, and TNFα was measured in cell culture supernatants using a Luminex Multiplex detection system (EMD Millipore). For each sample, data were determined as follows: (stimulated)−(unstimulated) = total (pg/mL) for each cytokine (mean of duplicate determinations) to show antigen-specific cytokine production.

### Serum ELISA for tick and αGal-specific IgE and MCPT-1 quantification

Total serum IgE and tick-specific IgE were determined by ELISA as previously described (14). For total serum titers, Costar high-binding plates (Corning) were coated with 0.83 μg/mL unlabeled antimouse IgE in PBS (Southern Biotechnologies). For tick-specific ELISA, high-binding plates were coated with tick extract at a concentration of 10 μg/mL in PBS. PBS containing 1% BSA, which does not have αGal (26, 27), was used to block non-specific binding to plates and as a sample buffer. The serum was diluted at 1:100 for IgE and serially titrated in threefold increments. HRP-labeled antimouse IgE (Southern Biotechnologies) served as the detection antibody, and the assay was developed using tetramethylbenzidine (BD Pharmingen) with 2 N H_2_SO_4_ as the stop solution. Total IgE antibody titers were quantified through a standard curve obtained using unlabeled IgE (Southern Biotech) with a detection limit of 0.07 μg/mL and a detection range of 0.34–250 μg/mL. Tick-specific IgE OD values were calculated as arbitrary units using a standard curve with pooled sera (starting at 1:50 and followed by threefold dilutions) from previous experiments. Alpha-gal IgE was detected by Luminex using a modified version of the Milliplex MAP mouse IgE single plex magnetic bead kit (Millipore Sigma, MGAMMAG-300E), where the kappa PE was replaced by αGal conjugated with biotin followed by streptavidin PE. Briefly, mouse sera (diluted 1:100 in assay buffer) was incubated with antimouse IgE beads for 1 h at room temperature (RT). After washing, beads were incubated with 25 μg/mL biotinylated αGal (Dextra Laboratories, NGB1334) for 18 h at 4°C, washed again, incubated with 25 μL of streptavidin-PE (Millipore Sigma, L-SAPE12) for 30 min at RT, washed, and resuspended in 150 μL of PBS. Samples were run through a Luminex XMAP INTELLIFLEX in the Flow Cytometry Core at the University of Virginia. Data are expressed as the PE mean fluorescence intensity (MFI) of the samples minus the PE MFI of the blank. Mouse sera from sensitized wild-type mice that produce αGal and thus do not make IgE against αGal were used as negative controls. For αGal-specific IgG1 and IgG2b ELISAs, high-binding plates were coated with 3 μg/mL αGal polymer (GlycoTech) in PBS, and serum was diluted at 1:50 and 1:200. Detection antimouse IgG1 and IgG2b antibodies were directly conjugated with HRP and diluted 1:1,000 in 1% BSA PBS, with a detection limit of 0.04 OD value and a detection range of 0.08–0.9 OD values. Mouse MCPT-1 serum levels diluted at 1:3 and serially titrated in threefold increments were measured by ELISA with a detection limit of 46.9 pg/mL and a detection range of 78.125–5,000 pg/mL according to the manufacturer's protocol (LSBio). All ELISA analyses included a standard, blank, and samples in duplicate and were read at 450 nm using a BioTek plate reader. The data were analyzed using curve-fitting software MyAssays (MyAssays.com) with a four-parameter algorithm to generate the standard curve, and after subtracting background absorbance from all data points and taking dilution factors into account, the concentrations of unknown samples were compared to the standard curve.

### Flow cytometry

Mouse peripheral blood and inguinal and mesenteric lymph nodes were collected 90 min after oral gavage with beef extract and analyzed for frequencies of CD45 ^+ ^CD49b ^+ ^Fc*ε*R1 ^+ ^c-kit^−^ basophils, CD45 ^+ ^Fc*ε*R1 ^+ ^c-kit^+^ mast cells, or CD45 ^+ ^CD103 ^+ ^CD11c ^+ ^F4/80^−^ dendritic cells (DCs) as previously described (14). Cells were stained with CD45-APC (Clone 104; eBioscience), CD49b-PerCP-Cy5.5 (Clone DX5; BioLegend), Fc*ε*R1alpha-FITC (Clone MAR-1; BioLegend), c-kit-PE-Cy7 (Clone 2B8; eBioscience), CD103-BV711 (Clone M290; BD Biosciences), CD11c-PE (Clone HL3; BD Biosciences), and F4/80-PerCP-Cy5.5 (Clone T45-2342; BD Biosciences). In stains as indicated, cells were also stained with CD200R (OX2R; CD200R1)-PE (Clone OX-110; BioLegend) and CD41-Brilliant Violet 421 (Clone WMReg30; BD Biosciences). Cell viability was determined using LIVE/DEAD Aqua (Invitrogen), and doublets were excluded based on forward scatter and pulse width. Samples were fixed in 1% paraformaldehyde, washed, acquired on an Attune Nxt cytometer, and analyzed using FlowJo software version 10.8.2 (Tree Star). Gates were determined using fluorescence minus one staining control.

### Basophil reactivity to αGal

Heparinized blood from individual mice was obtained, and the resulting cells were cultured in basophil culture medium (5% AB serum RPMI supplemented with penicillin/streptomycin and 1 mM L-glutamine) in 96-well V-shaped plates for 1.5 h at 37°C with or without 10 μg/mL cetuximab as previously described (14). The cells were then spun down, and the culture supernatant was collected and frozen at −20°C. Histamine levels were subsequently measured by a competitive ELISA kit (Enzo Life Science) in the supernatant according to manufacturer's instructions. Fold increase was calculated as cetuximab divided by a non-stimulated sample for each mouse. All plates were read at 450 nm using a BioTek plate reader.

### Statistical analysis

Statistics were determined using Prism software v10 (GraphPad Software, Boston, MA). To assess differences between groups, an unpaired, two-tailed *t*-test or the non-parametric Mann–Whitney test was used. Error bars shown in each figure indicate the mean ± SEM. Significance was defined as *P*-values ≤ 0.05, and the significance levels are stated in the figure legends. All statistical differences are labeled on the graphs.

## Results

### Prophylactic treatment with αGal glycoprotein-containing nanoparticles reduces sensitization to the αGal oligosaccharide

Previously, we demonstrated that PLG NPs encapsulating protein allergens imitate the size and charge of apoptotic cellular debris and directly associate with APCs, leading to a reduction in the secretion of Th2 cytokines (28, 29). We sought to determine whether NPs containing αGal glycoprotein would induce a level of tolerance sufficient to prevent sensitization to αGal when NPs were administered prophylactically to an established mouse model of cutaneous sensitization to αGal (14). This mouse model uses the proallergic adjuvant effects of lone star tick protein extracts and the glycoprotein αGal-BSA to reliably induce αGal-specific IgE production in mice deficient in αGal (AGKO). NP-αGal and, as controls, containing human serum albumin alone (NP-HSA) were generated with a diameter of ∼500 nm and a zeta potential of ∼−40 mV as previously described (28). To test the effects of prophylactic treatment with NPs on immune cells responding to cutaneous αGal exposure, two doses of allergen-encapsulating NPs or an equivalent volume of PBS were intravenously delivered 2 weeks apart to AGKO mice (Figure 1A). AGKO mice were subsequently given a series of intradermal injections with lone star tick protein extract and αGal-BSA prior to the intragastric challenge with αGal-containing beef extract as previously described (14). Unsensitized controls consisted of naïve mice. On day 35, mice that were sensitized after PBS i.v. injection displayed significant reactions to the intragastric beef extract challenge, as measured by increased serum MCPT-1 levels (Figure 1B) and histamine levels released by circulating blood basophils after *in vitro* stimulation with cetuximab, a monoclonal antibody containing αGal moieties in the Fab portion of its heavy chain (4, 30) (Figure 1C). As expected, neither MCPT-1 in serum nor histamine released by cetuximab-stimulated basophils was detected from naïve mice, demonstrating that sensitization to αGal through the skin contributes to a hypersensitivity response following meat consumption. Mice that were sensitized after treatment with NPs that contained αGal (NP-αGal) showed significantly reduced levels of histamines and MCPT-1 compared to mice treated with PBS following the intragastric beef extract challenge, supporting reduced sensitization to αGal in mice that were prophylactically treated with NP-αGal. Mice that were intradermally exposed to lone star tick protein extract and αGal-BSA after treatment with NPs that contained the irrelevant HSA protein (NP-HSA) showed increased levels of histamines released by basophils, similar to the levels observed from mice treated with PBS and significantly higher compared to NP-αGal-treated mice (Figure 1C). Increased levels of MCPT-1 were found in sera from mice treated with NP-HSA following the intragastric beef extract challenge, trending higher than NP-αGal-treated mice but significantly lower compared to mice treated with PBS (Figure 1B).

Prompted by our *in vivo* data that prophylactic treatment with NP-αGal significantly reduced the levels of MCPT-1 and histamines, we analyzed sera from AGKO mice administered with NPs or PBS and then immunized with tick protein extract and αGal-BSA for levels of total IgE and tick antigen- and αGal-specific IgE by ELISA. First, we confirmed that total, tick-specific, and αGal-specific IgE levels were induced in sera from sensitized mice treated with PBS compared to naïve controls (Figure 1D). As expected, these IgE antibodies were also induced in sensitized mice treated with control NP-HSA. Total IgE levels were induced in sensitized mice treated with NP-αGal, similar to PBS- and NP–HSA-treated mice, with tick-specific IgE levels significantly reduced relative to PBS- and NP-HSA-treated groups. We further found that αGal-specific IgE levels were significantly reduced in NP-αGal-treated mice, with 36% of mice expressing αGal-specific IgE compared to the PBS- and NP-HSA-treated groups that had 82% and 80% of mice expressing αGal-specific IgE, respectively (Figure 1D, right panel). Levels of αGal-specific IgG1 were also significantly reduced in NP-αGal-treated mice compared to PBS treatment, while no differences in the levels of IgG2b to αGal were observed between the groups of sensitized mice (Figure 1D). The total numbers of germinal center B cells (B220 ^+ ^GL-7 ^+ ^CD95^+^) and IgE^+^ plasma cells (B220^−^CD138^+^) within the skin-draining inguinal lymph nodes increased in all groups of sensitized mice compared to naïve controls, with a trend, although not statistically significant, toward fewer numbers of both cell types measured in NP-αGal-treated animals (Supplementary Figure S1). These results suggest that the mechanism by which MCPT-1 and histamine release levels were reduced in mice prophylactically treated with NP-αGal and orally challenged with beef extract was partially through reduced αGal-specific IgE production.

### Prophylactic treatment with αGal glycoprotein-containing nanoparticles reduces basophil frequency and activation in mesenteric lymph nodes

**Figure 2:**
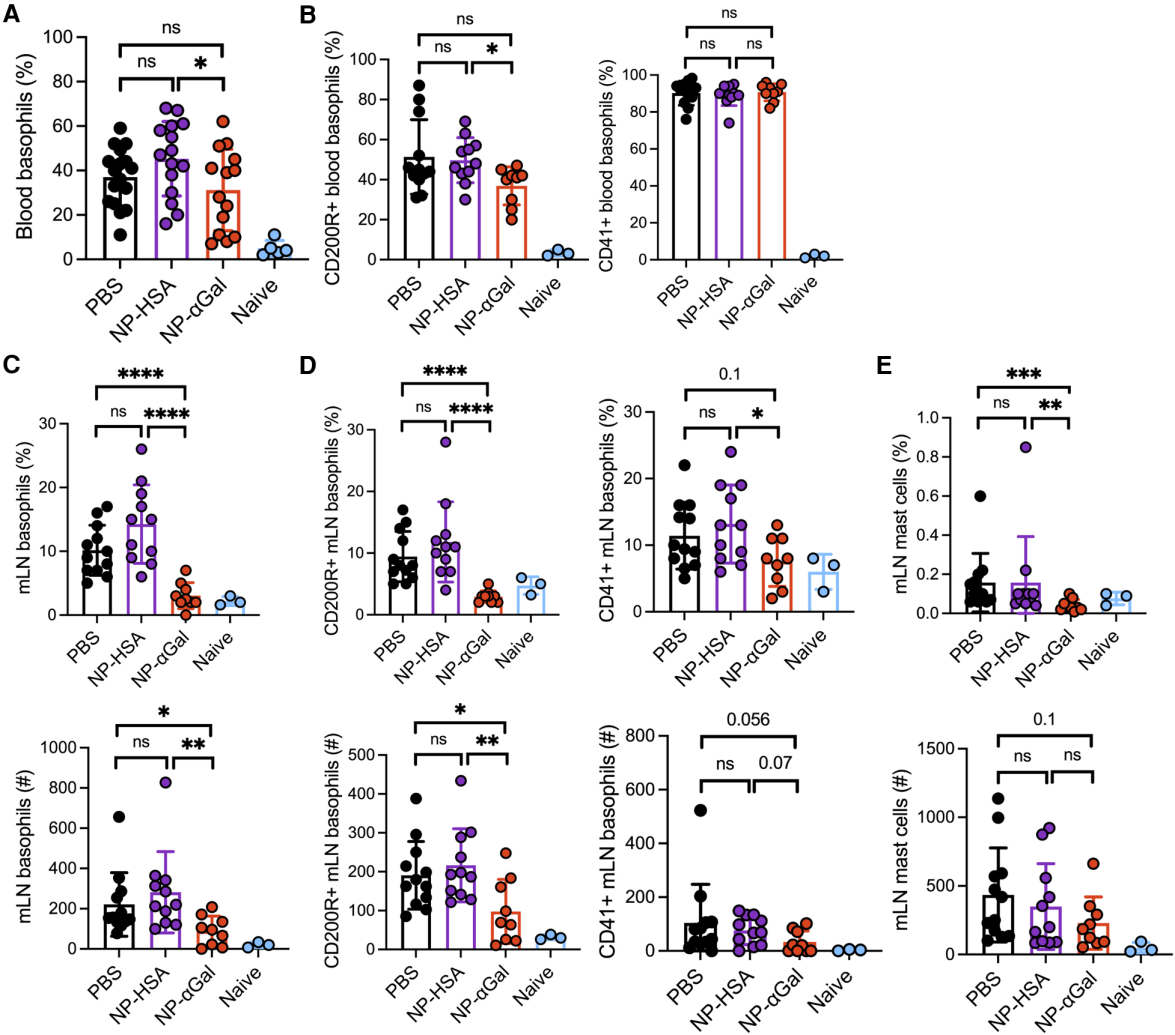
Prophylactic administration of NP-αGal reduces the frequency and activation of basophils in the mesenteric lymph nodes of mice following an intragastric challenge with beef extract. (**A,B**) Frequency and activation of circulating CD45 ^+ ^CD49b ^+ ^Fc*ε*R1 ^+ ^IgE ^+ ^c-kit^−^ basophils in peripheral blood from mice at 90 min after oral gavage measured by flow cytometry. (**C,D**) Frequencies (top), numbers (bottom), and activation of basophils in mesenteric lymph nodes of the same mice as in panel (**A,B**). (**E**) Frequencies (top) and numbers (bottom) of Fc*ε*R1 ^+ ^c-kit^+^ mast cells in mesenteric lymph nodes. The results shown are representative of two independent experiments. All data are expressed as mean ± SEM. *P* = *0.05, ***0.001, and ****0.0001, with an unpaired, two-tailed *t*-test.

In murine models of food allergy, basophils play a significant role in the sensitization phase (31–33). To determine the impact of prophylactic administration of NPs on basophils, we sensitized AGKO mice with tick extract plus αGal-BSA after treatment with NP-αGal, NP-HSA, or PBS and assessed the frequency and activation of basophils by flow cytometry. We focused on circulating and mesenteric lymph node basophils that drain the gastrointestinal tract because of the oral challenge. Analysis of peripheral blood basophils (CD45 ^+ ^CD49b ^+ ^Fc*ε*R1 ^+ ^IgE ^+ ^c-kit^−^) from all groups of sensitized mice showed that the percentages of basophils significantly increased after the intragastric challenge with beef extract compared to naïve controls, with lower basophil frequencies found in mice treated with NP-αGal compared to those treated with NP-HSA (Figure 2A; Supplementary Figure S2). Sensitized mice also exhibited increased frequencies of blood basophils that expressed the basophil activation markers CD200R and CD41 (34–36) following an oral challenge with beef extract regardless of prophylactic treatment (Figure 2B). However, mice treated with NP-αGal showed reduced frequencies of blood basophils with increased expression of CD200R but not CD41 compared to NP-HSA treatment. The groups of sensitized mice that were prophylactically treated with NP-HSA and PBS also showed that the percentages and the numbers of basophils significantly increased in the mesenteric lymph nodes compared to mice treated with NP-αGal (Figure 2C). Moreover, the percentages and the total numbers of basophils in the mesenteric lymph nodes that expressed CD41 and CD200R were similarly increased in the groups of mice treated with NP-HSA and PBS compared to those treated with NP-αGal (Figure 2D). Mast cells are found throughout the gastrointestinal tract and are increased in food-allergic participants (37–40). As expected, increased percentages and numbers of Fc*ε*R1 ^+ ^c-kit^+^ mast cells in the mesenteric lymph nodes were found in sensitized mice prophylactically treated with NP-HSA and PBS compared to naïve controls (Figure 2E). Mice treated with NP-αGal showed reduced percentages of mast cells and fewer numbers in mesenteric lymph nodes compared to those treated with NP-HSA, although these differences did not reach significance. Taken together, these results demonstrate that tick-induced sensitization to αGal promotes increased intestinal basophils and mast cells, which are reduced by prophylactic NP-αGal treatment. Decreased activation of mesenteric lymph node basophils (Figure 2D) and serum levels of MCPT-1 (Figure 1B) in sensitized mice treated with NP-αGal suggests that NP-αGal treatment also reduces degranulation of basophils and mast cells in response to the intragastric challenge with αGal-containing beef extract.

### Prophylactic treatment with αGal glycoprotein-containing nanoparticles reduces Th2 cytokine production

**Figure 3:**
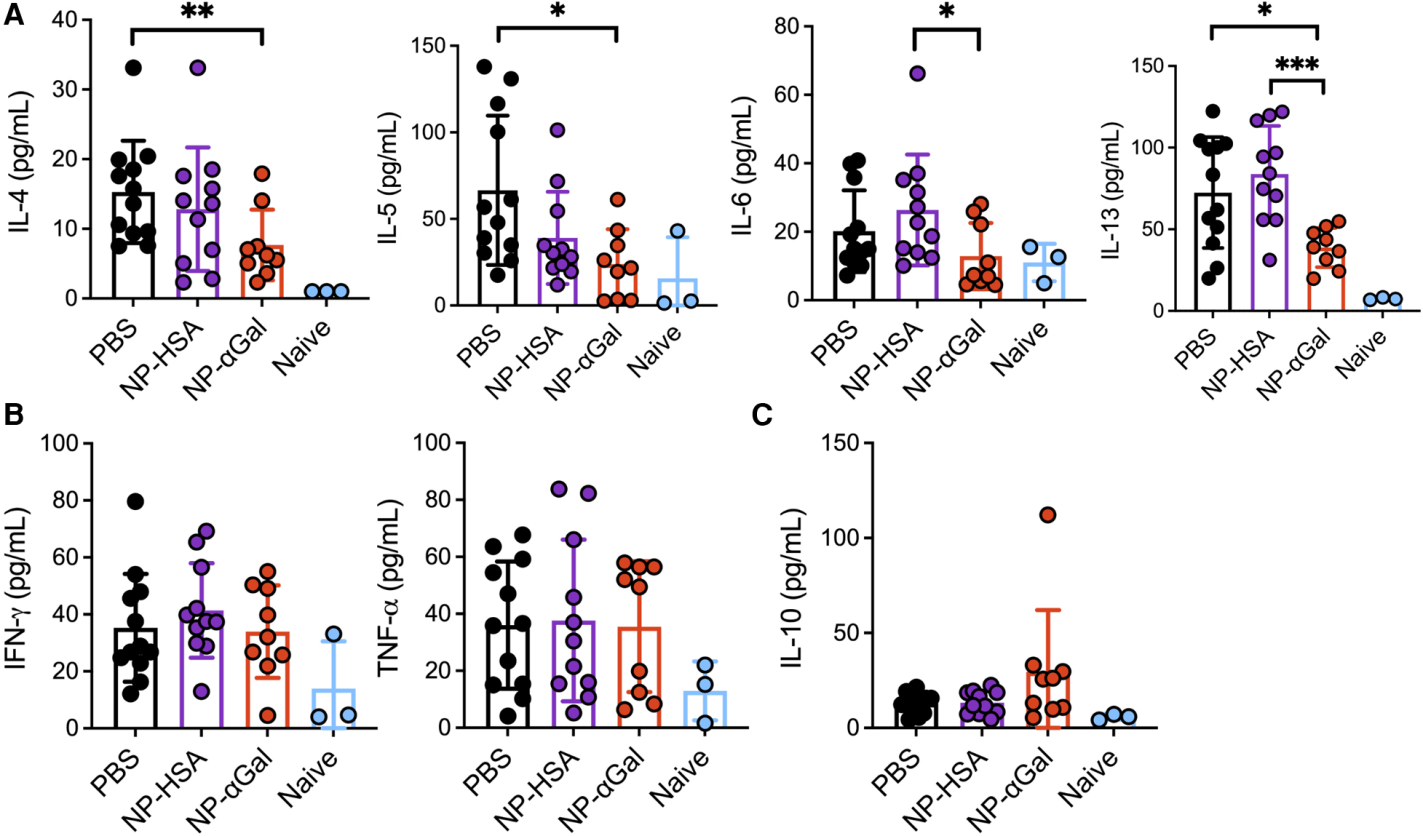
Prophylactic administration of NP-αGal reduces IL-4, IL-5, IL-6, and IL-13 and increases IL-10. (**A–C**) Cytokine secretion in culture supernatants from splenocyte recall assays to cetuximab measured by Luminex multiplex. The results shown are representative of two independent experiments. All data are expressed as mean ± SEM. *P* = *0.05, **0.01, and ***0.001, with a non-parametric Mann–Whitney test.

Our group and others have demonstrated that PLG NPs given intravenously to deliver protein cargo to antigen-presenting cells in the spleen reduce Th2 cell activation (17, 18). Thus, we investigated the effects of prophylactic administration of NPs on allergic cytokine production by splenic cells following stimulation with αGal. Splenocytes from mice that were sensitized after treatment with NP-αGal, NP-HSA, or PBS were obtained and stimulated with cetuximab to induce αGal-dependent cytokine production and assessed 3 days later for the presence of Th1, Th2, and regulatory T-cell cytokines in the cell culture medium. Splenocytes from naïve mice served as negative controls. Analysis of the response to cetuximab in recall assays of splenic cells from NP-αGal-treated mice but not mice treated with NP-HSA or PBS showed a significant reduction in the secretion of Th2 cytokines IL-4, IL-5, IL-6, and IL-13 (Figure 3A). No effects were detected in the secretion of Th1 cytokines IFN-*γ* and TNF-α regardless of the NP treatment group compared to PBS controls (Figure 3B). Antigen-specific T-cell suppression through the production of IL-10 contributes to the development of oral tolerance (41, 42). Interestingly, increased IL-10 secretion from recall assays of splenocytes of NP-αGal-treated mice but not mice treated with NP-HSA or PBS was found, suggesting that αGal-dependent induction of IL-10 secretion contributes to reduced sensitization to αGal (Figure 3C). Taken together, these results show that prophylactic NP-αGal treatment blocks the secretion of Th2 cytokines associated with food allergy.

### Therapeutic treatment with αGal glycoprotein-containing nanoparticles reduces allergic responses to αGal

**Figure 4:**
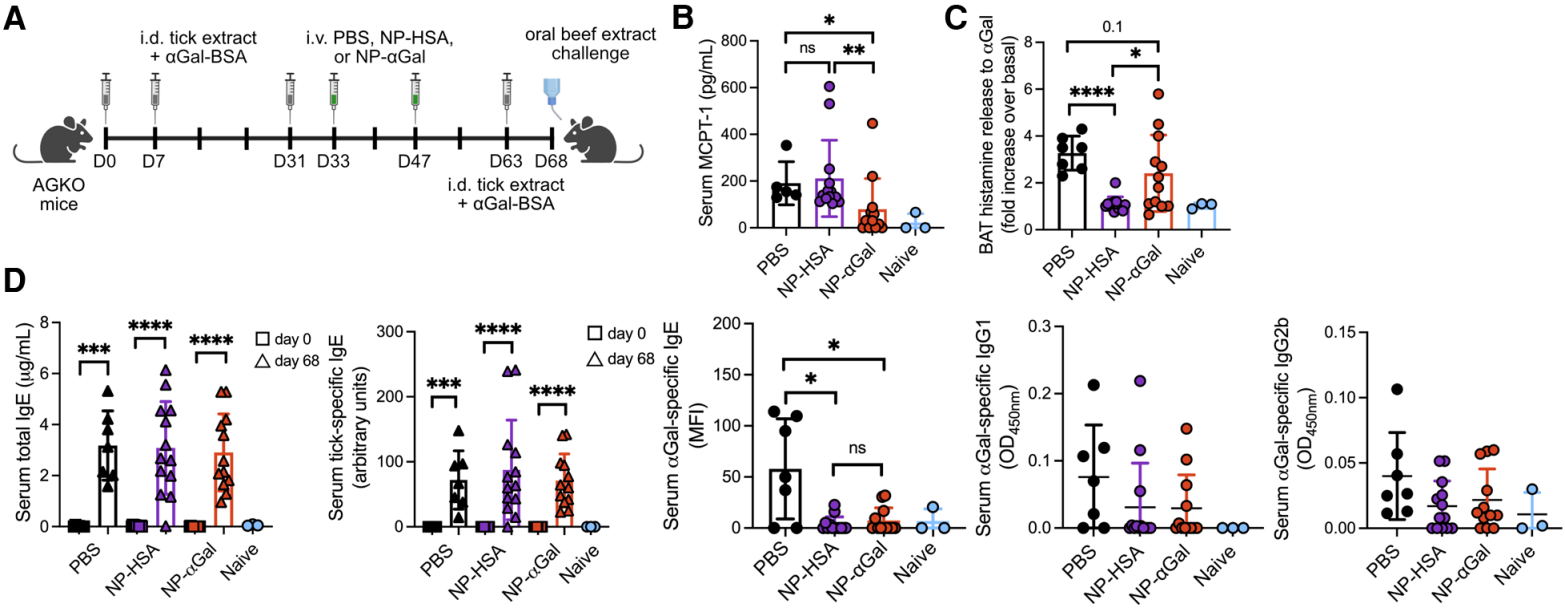
Therapeutic administration of NP-αGal reduces hypersensitivity responses in mice following an intragastric challenge with beef extract. (**A**), Schematic of therapeutic NP treatment. AGKO mice received intradermal injections (i.d.) of whole-body protein extract prepared from lone star seed ticks supplemented with 50μg αGal-BSA on day 0 and identically prepared booster injections on days 7 and 31. On days 33 and 47, mice were intravenously given NP-αGal–HSA or control NP-HSA and, 2 weeks later, were given a booster of tick extract. On day 68, mice received an intragastric challenge with beef extract and were analyzed 1.5 h later. (**B**) Concentration of MCPT-1 in the sera of mice after a beef extract challenge measured by ELISA. (**C**) Fold increase in histamine released from the basophil activation test (BAT) with cetuximab measured by ELISA. (**D**) Serum levels of total IgE, tick-specific IgE, and αGal-specific IgE, IgG1, and IgG2b from mice on day 68 measured by ELISA or Luminex. All data are expressed as mean ± SEM. *P* = *0.05, and ****0.0001, or ns = not significant, with an unpaired, two-tailed *t*-test.

The therapeutic efficacy of PLG NPs to suppress allergen-specific immune responses has been shown in murine models of airway and peanut allergy (17–19). Thus, we sought to determine whether therapeutically administered NPs containing αGal would reduce allergic reactivity in AGKO mice with established sensitization to αGal. Mice were sensitized intradermally with tick extract plus αGal-BSA and received two intravenous doses of NPs or PBS; 2 weeks later, they were given a booster with tick extract, followed 4 days after that with an intragastric challenge with beef (Figure 4A). On day 68, mice that were sensitized and treated with PBS displayed significant reactions to the intragastric beef extract challenge, as measured by increased serum MCPT-1 levels (Figure 4B) and histamine levels released by circulating blood basophils after *in vitro* stimulation with cetuximab (Figure 4C). As expected, neither serum MCPT-1 levels nor histamine released by cetuximab-stimulated basophils were detected in naïve mice. Mice sensitized and treated with NP-αGal showed significantly reduced MCPT-1 levels and lower histamine levels (although not statistically significant) compared to mice treated with PBS following the intragastric beef extract challenge. MCPT-1 levels were also reduced in mice treated with NP-αGal relative to those in control NP-HSA-treated mice (Figure 4B). Mice treated with NP-HSA showed significantly lower histamine levels compared to PBS- and NP-αGal-treated mice (Figure 4C). However, basophils activated with cetuximab showed no increase in histamine release over unstimulated basophils from the peripheral blood of mice treated with NP-HSA, which resulted from greater basal levels of histamine released from unstimulated basophils (Supplementary Figure S3), supporting reduced hypersensitivity to αGal in mice that were therapeutically treated with NP-αGal. As expected, total and tick-specific IgE levels were induced in sera from all groups of sensitized mice regardless of therapeutic treatment compared to naïve controls (Figure 4D). We further found that αGal-specific IgE levels were significantly reduced in mice treated with NP-αGal or NP-HSA compared to those in the PBS-treated and naïve groups. No differences in the levels of αGal-specific IgG1 or IgG2b were found between the sensitized groups (Figure 4D).

**Figure 5:**
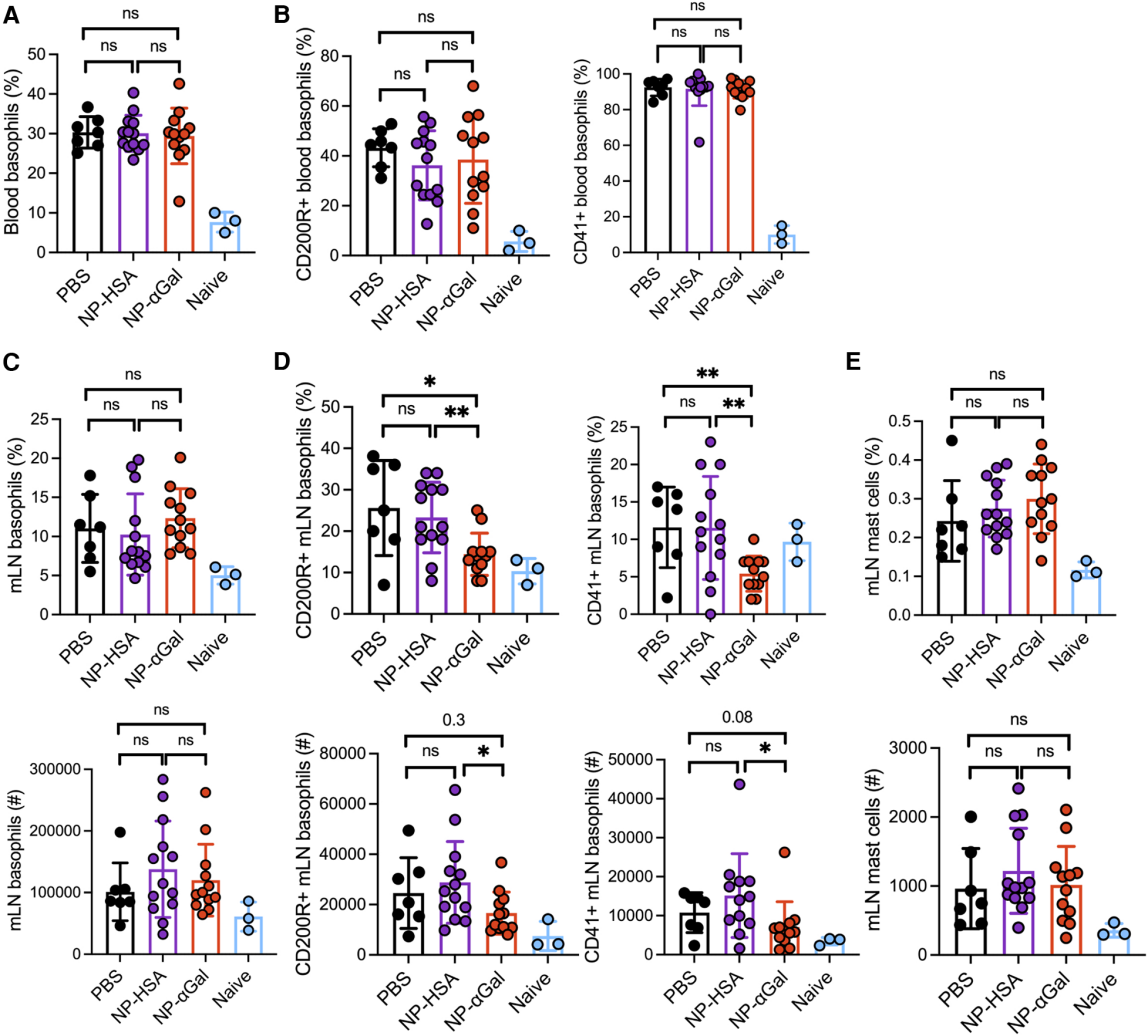
Therapeutic administration of NP-αGal reduces the activation of basophils in the mesenteric lymph nodes of mice following an intragastric challenge with beef extract. (**A,B**) Frequencies of circulating CD45 ^+ ^CD49b ^+ ^Fc*ε*R1 ^+ ^IgE ^+ ^c-kit^−^ basophils and activation by CD200R and CD41 expression in peripheral blood from mice at 90 min after beef gavage measured by flow cytometry. (**C,D**) Frequencies (top), numbers (bottom), and activation of basophils in mesenteric lymph nodes of the same mice as in panel (**A,B**). (**E**) Frequencies (top) and numbers (bottom) of Fc*ε*R1 ^+ ^c-kit^+^ mast cells in mesenteric lymph nodes. All data are expressed as mean ± SEM. *P* = *0.05, and **0.01, with an unpaired, two-tailed *t*-test.

An increase in peripheral blood basophils expressing activation markers CD200R and CD41 was observed in all groups of sensitized mice regardless of therapeutic treatment after intragastric challenge with beef extract compared to those in naïve controls (Figure 5A,B). Analysis of basophil frequencies in mesenteric lymph nodes from sensitized mice showed no differences between treatment groups (Figure 5C). However, sensitized mice therapeutically treated with NP-αGal demonstrated reduced percentages and numbers of basophils expressing CD41 and CD200R compared to mice treated with NP-HSA and PBS (Figure 5D). No differences in the percentages and numbers of mast cells in the mesenteric lymph nodes were found in mice among the treatment groups. These results indicate that while therapeutic administration of NP-αGal using this treatment regimen and the experimental timeline does not affect basophil or mast cell frequencies upon an oral challenge, basophil activation was significantly reduced in mesenteric lymph nodes.

**Figure 6:**
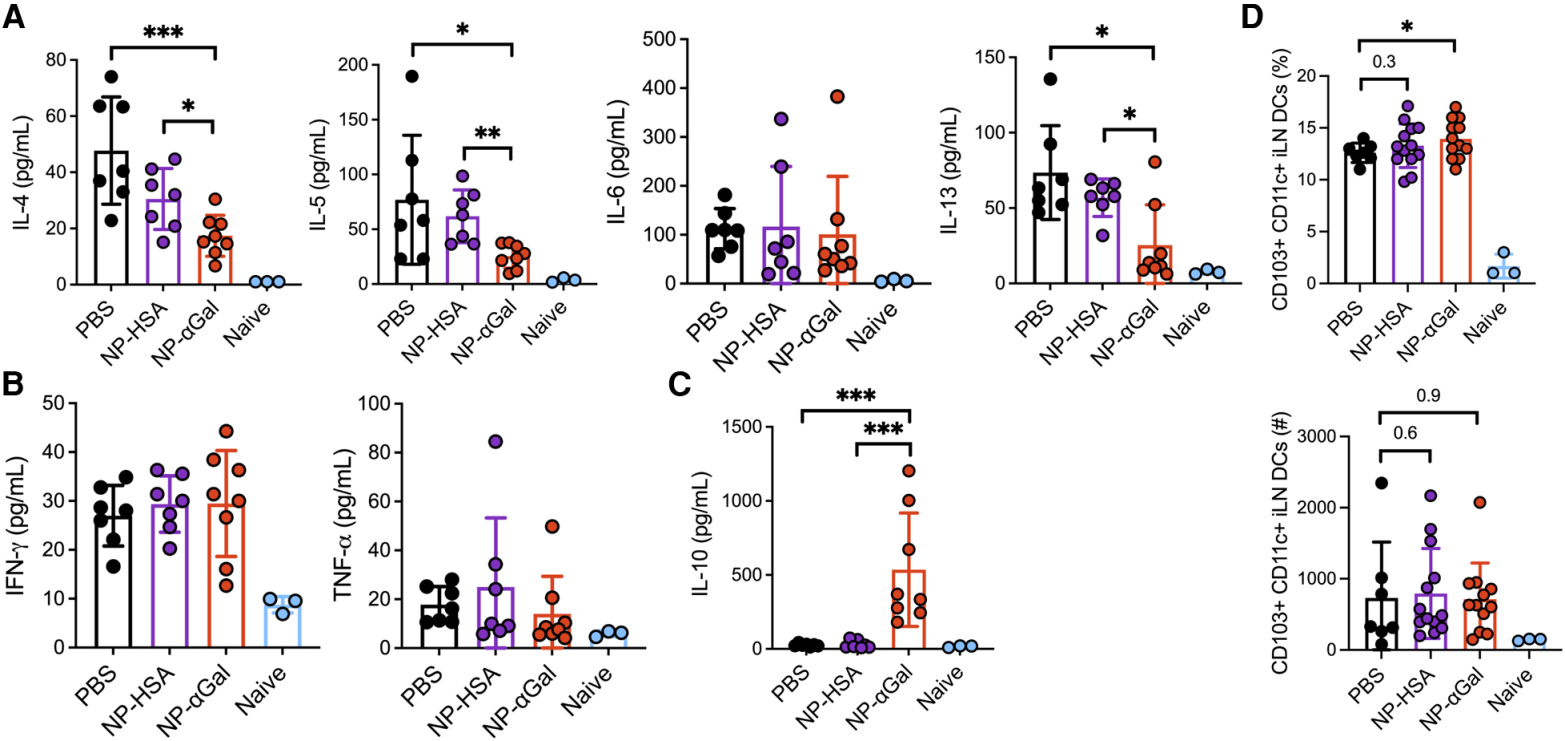
Therapeutic administration of NP-αGal reduces IL-4, IL-5, IL-6, and IL-13 and increases IL-10. (**A–C**) Splenocytes harvested on day 68 from all groups of sensitized mice therapeutically administered NPs were stimulated with cetuximab, a source of αGal, and cytokine secretion in culture supernatants from splenocyte recall assays was measured by Luminex multiplex. (**D**) Frequencies of CD103 ^+ ^CD11c ^+ ^F4/80^−^ dendritic cells in skin-draining inguinal lymph nodes of the same mice as in panels (**A–C**) measured by flow cytometry. All data are expressed as mean ± SEM. *P* = *0.05, **0.01, and ***0.001, with a non-parametric Mann–Whitney test (**A–C**) or an unpaired, two-tailed *t*-test (**D**).

*Ex vivo* analysis of the response of spleen cells from control and NP-treated mice demonstrated reduced αGal-specific IL-4, IL-5, and IL-13 production following therapeutic administration of NP-αGal (Figure 6A). Cetuximab stimulation of splenocytes from NP-αGal-treated mice did not increase IFN*γ* and TNFα production (Figure 6B) but significantly increased IL-10 production (Figure 6C) compared to controls and NP-HSA-treated mice. CD103^+^ DCs at sites of allergen drainage play a critical role in the generation of natural oral tolerance, and prior studies have suggested that they also mediate desensitization in food-allergic oral immunotherapy participants (43–45). Thus, we evaluated sensitized mice treated with NPs or PBS, followed 2 weeks later by an intradermal booster with tick extract, and 4 days after that, we assessed the frequencies of CD103^+^ DCs in the skin-draining inguinal lymph nodes. The percentages of CD103 ^+ ^CD11c ^+ ^F4/80^−^ DCs and numbers (although not statistically significant) were increased in mice treated with NPs compared to those in PBS controls (Figure 6D), suggesting that therapeutic administration of NPs may induce DCs away from a Th2 skewing phenotype. Reduced levels of IL-4, IL-5, and IL-13, known to promote IgE class switching, and increased levels of IL-10 produced by splenocytes from NP-αGal-treated mice following cetuximab stimulation suggest that the effects of NP-αGal were sufficient to suppress allergic cytokine production.

## Discussion

Using an established mouse model of tick-induced IgE sensitization to the mammalian oligosaccharide αGal, we have investigated intravenous administration of glycoprotein allergen-encapsulating NPs for their ability to attenuate allergic responses after an intragastric challenge with αGal-containing beef extract. Our studies demonstrate that these glycoprotein-encapsulating NPs may be used to prophylactically prevent tick-induced sensitization to the carbohydrate αGal while providing insight into the immune pathways involved in desensitization to αGal by NPs. No prior studies have investigated the pro-tolerogenic immunological changes occurring following carbohydrate antigen-specific reprogramming by NPs. Our current work shows that NP-αGal prophylactic treatment alters immune pathways involving T cells, basophils, and mast cells. When delivered prior to sensitization, NPs containing αGal-HSA effectively reduced serum MCPT-1 levels and αGal-specific basophil histamine levels, which corresponded to reduced basophil frequencies and activation of basophils in mesenteric lymph nodes. Further, mast cell frequency trended downward in the mesenteric lymph nodes. Alpha-gal-specific stimulation of splenocytes from mice prophylactically treated with NP-αGal significantly reduced the production of IL-4, IL-5, and IL-13 and increased the production of regulatory cytokine IL-10 without skewing toward a Th1 phenotype. These findings suggest that NP-αGal treatment can reprogram cytokine production in the spleen, a central tolerogenic organ, to prevent elevated Th2 responses, subsequently leading to reduced αGal-specific IgE and IgG1 production. The effects of NP-αGal on Th2 cytokine responses are consistent with our previous report showing that IgE sensitization to αGal depends CD4^+^ T cell help (14). In AGS patients, αGal-specific IgE production is associated with increased levels of specific IgG1 antibodies (46–48). However, the significance of αGal-specific IgG1 in the development of AGS has not been clarified yet. The role of CD4^+^ T cells has not yet been definitively determined in humans with AGS nor in another mouse model of AGS involving subcutaneous sensitization of AGKO mice with tick salivary gland extract (15).

Analysis of sensitized mice therapeutically treated with NPs revealed that NP-αGal significantly reduced serum MCPT-1 levels and histamines released by basophils, although the latter was not statistically significant. Moreover, activation but not frequencies of basophils in mesenteric lymph nodes was reduced in mice treated with NP-αGal. No changes in the frequencies of mast cells in the mesenteric lymph nodes of mice were detected. The modest effects of NP-αGal on basophil and mast cell frequencies when given therapeutically compared to prophylactically suggest that therapeutic administration of NPs may not affect the expansion of these cell types in the mesenteric lymph nodes when pre-existing IgE antibodies are bound to Fc*ε*R. We also revealed that therapeutic delivery of NP-αGal to sensitized mice suppressed the production of IL-4, IL-5, and IL-13 and increased the production of immunosuppressive cytokine IL-10 from splenocytes stimulated with αGal. Allergen immunotherapy has been shown to induce IL-10 following grass pollen subcutaneous immunotherapy, sublingual immunotherapy (49), and house dust mite (HDM) subcutaneous immunotherapy (50, 51). Generation of T regulatory (Treg) cells that produce IL-10 *in vivo* after tolerance induction with oral antigens has been reported (52, 53). Thus, increased production of IL-10 from splenocytes stimulated with αGal may suggest that therapeutic administration of NP-αGal to sensitized mice induces a T regulatory cell subset that plays a role in oral tolerance. Using a mouse model of egg allergy, we recently showed that protein allergen-encapsulating NPs can reprogram pathogenic allergen-specific Th2 cells toward a T regulatory phenotype in the small intestine lamina propria (21). Interestingly, a trend increase in the frequency of CD103^+^ DCs was found in the skin-draining lymph nodes of mice treated with NP-αGal. CD103^+^ DCs isolated from the mesenteric lymph nodes of mice and humans induce the differentiation of naïve T cells into Treg cells (43, 54, 55). Future experiments devoted to assessing the effects of NP-αGal to reprogram carbohydrate antigen-specific T cells in the gastrointestinal tract will clarify this point.

While NP-αGal treatment is effective at inducing tolerance to αGal and reducing allergic reactivity in sensitized mice by reducing αGal-specific Th2 cytokine production, our data demonstrate that NPs may have a combination of both non-specific and antigen-specific effects in the therapeutic setting. Control NP-HSA given therapeutically increases MCPT-1; however, serum αGal-specific IgE levels are reduced. The differential responses observed with the NP-HSA likely represent some combination of tick antigen-specific IgE bound to mast cells that induce activation by IgE aggregation with multivalent tick antigens that persist in the 5 days following the booster with tick extract. The antigen-specific responses observed with NPs are typically associated with antigen-presenting cells interacting with T cells (56). The decrease in αGal-specific IgE from the NP-HSA, which does not contain the αGal antigen, may arise from a context-dependent bystander effect. In the therapeutic setting, B cells specific to αGal may be abundant at the time of NP administration and associate with the NPs, altering their phenotype or function. Previous studies have demonstrated that NPs can associate with B cells and innate immune cells and alter their phenotype (57–60). Moreover, NPs associated with B cells have been reported to modulate T-cell responses (60). Therefore, NP-mediated reprogramming of immune cells independent of antigen may contribute to less production of αGal-specific IgE in the therapeutic setting.

While these studies have focused on the effects of NPs encapsulating αGal when linked to a protein to attenuate allergic responses to αGal, αGal can also be linked to lipids (10). Invariant natural killer T (iNKT) cells recognize lipid antigens and can produce cytokines traditionally associated with Th1, Th2, and regulatory populations. Further study is necessary to determine the ability of αGal glycolipids to activate iNKT cells and promote the production of αGal-specific IgE. Also unknown is whether treatment with αGal glycoprotein-containing NPs would be sufficient to desensitize recipients sensitized with the αGal glycolipid. Further studies are needed to assess whether NPs containing the αGal glycoprotein, αGal glycolipid, or αGal carbohydrate alone would be capable of tolerizing recipients sensitized with αGal glycoprotein or αGal glycolipid.

Current treatment modalities for patients with AGS are limited to preventing new tick bites and avoidance of mammalian meats and mammalian-derived food products and drugs. However, allergen avoidance leaves patients susceptible to accidental allergen exposures and economic and social consequences related to avoidance-imposed lifestyle changes (11). Alternatively, recent reports have shown that patients with AGS who underwent oral immunotherapy with red meat became tolerant to red meat (12, 13, 61). Oral immunotherapy for any allergen currently relies on continuous daily dosing, which, despite careful surveillance of young and old patients alike, results in high exposure to treatment-related adverse effects (12, 62). NPs offer multiple advantages over traditional vaccine delivery methods for desensitization. First, because of their size and surface charge, these NPs are selectively taken up by antigen-presenting cell populations. This enables the enhanced uptake of allergen loaded into these NPs by antigen-presenting cells. Second, because the allergen is encapsulated within these NPs, it is masked from circulating antibodies and immune cells. Therefore, treatment with allergens encapsulated within NPs confers a reduced likelihood of eliciting an allergic reaction relative to treatment with free allergens. In our studies, prophylactic treatment with only two doses of NP-αGal is sufficient to prevent sensitization to carbohydrate antigen-specific responses and reduce allergic burden following subsequent exposure to beef extract. Moreover, therapeutic administration of two doses of αGal-containing nanoparticles to mice sensitized to αGal had partial efficacy by reducing Th2 cytokine production, αGal-specific IgE production, and MCPT-1 release but did not reduce basophil activation or histamine release and αGal-specific IgG1 or IgG2b levels. Given these promising results, our work shows that NP-αGal can be successfully exploited to improve allergen-specific immunotherapy outcomes. However, subsequent studies are needed to address the therapeutic potential of additional NP-αGal doses on allergic reactivity and the long-lasting effects of NP treatment. Experiments comparing repeated intragastric challenges with beef extract to deglycosylated beef extract may help dissect non-specific and antigen-specific effects of NPs in the therapeutic setting and determine the durability of the induced tolerance. We anticipate that this tolerance could be sustained following repeated oral exposure to antigens, as we have previously demonstrated in a model of egg allergy with multiple oral egg challenges (21). Consistent with many immunotherapies, our findings reveal that some mice respond to NP treatment while others do not. Further studies to elucidate immune mechanisms of allergic desensitization by NPs may also provide novel insights into underlying causes for the variable responses to NP treatment.

In conclusion, these studies demonstrate the first therapeutic strategy using NPs to treat AGS, an understudied tickborne food allergy to mammalian meat. We show that prophylactic treatment with αGal glycoprotein-containing NPs reduces splenocyte Th2 cytokine production following stimulation with a different αGal-containing glycoprotein. αGal-glycoprotein NPs subsequently reduce total, tick-, and αGal-specific IgE levels in the blood while simultaneously reducing the reactivity of circulating basophils and mast cells. In addition, while the current studies demonstrate that these NPs hold prophylactic efficacy in preventing AGS formation, therapeutic treatment with αGal glycoprotein-containing NPs also reduces Th2 cytokines, αGal-specific IgE levels, and mast cell activity. To our knowledge, this is the first demonstration of immunological tolerance induction to an oligosaccharide. Our findings highlight the therapeutic potential of NP-αGal to reduce AGS in recipients with pre-existing disease.

## Data Availability

The original contributions presented in the study are included in the article/**Supplementary Material**; further inquiries can be directed to the corresponding authors.
