# Supplementary material for: Immunotherapy with biodegradable nanoparticles encapsulating the oligosaccharide galactose-alpha-1,3-galactose enhance immune tolerance against alpha-gal sensitization in a murine model of alpha-gal syndrome
Source: Front Allergy. 2024 Aug 9;5:1437523. doi: 10.3389/falgy.2024.1437523 (PMC11341473; doi:10.3389/falgy.2024.1437523)
Supplement: Supplementary file 1 [file Datasheet1.pdf]

frontiers

Supplementary Material

Immunotherapy with Biodegradable Nanoparticles Encapsulating the Oligosaccharide Galactose-Alpha-1,3-Galactose Enhance Immune Tolerance Against Alpha-Gal Sensitization in a Murine Model of Alpha-Gal Syndrome

Michael N. Saunders[1,2†], Claudia M. Rival[3,4†], Mahua Mandal[3,4], Kayla Cramton[3,4], Laila M. Rad[1], Katarzyna W. Janczak[5], Laura A. Williams[1], Amogh R. Angadi[1], Jessica J. O'Konek[5*], Lonnie D. Shea[1,6,7*], and Loren D. Erickson[3,4*]

[1]Department of Biomedical Engineering, University of Michigan, Ann Arbor, MI, United States

[2]Medical Scientist Training Program, University of Michigan, Ann Arbor, MI, United States

[3]Beirne Carter Center for Immunology Research, University of Virginia, Charlottesville, VA, United States

[4]Department of Microbiology, Immunology, and Cancer Biology, University of Virginia, Charlottesville, VA, United States

[5]Mary H. Weiser Food Allergy Center, Michigan Medicine, Ann Arbor, MI, United States

[6]Department of Chemical Engineering, University of Michigan, Ann Arbor, MI, United States

[7]Department of Surgery, University of Michigan, Ann Arbor, MI, United States

† These authors share first authorship

*** Correspondence:**
Loren D. Erickson
loren@virginia.edu

Lonnie D. Shea
ldshea@umich.edu

Jessica J. O'Konek
jjoz@umich.edu

Supplementary Figures

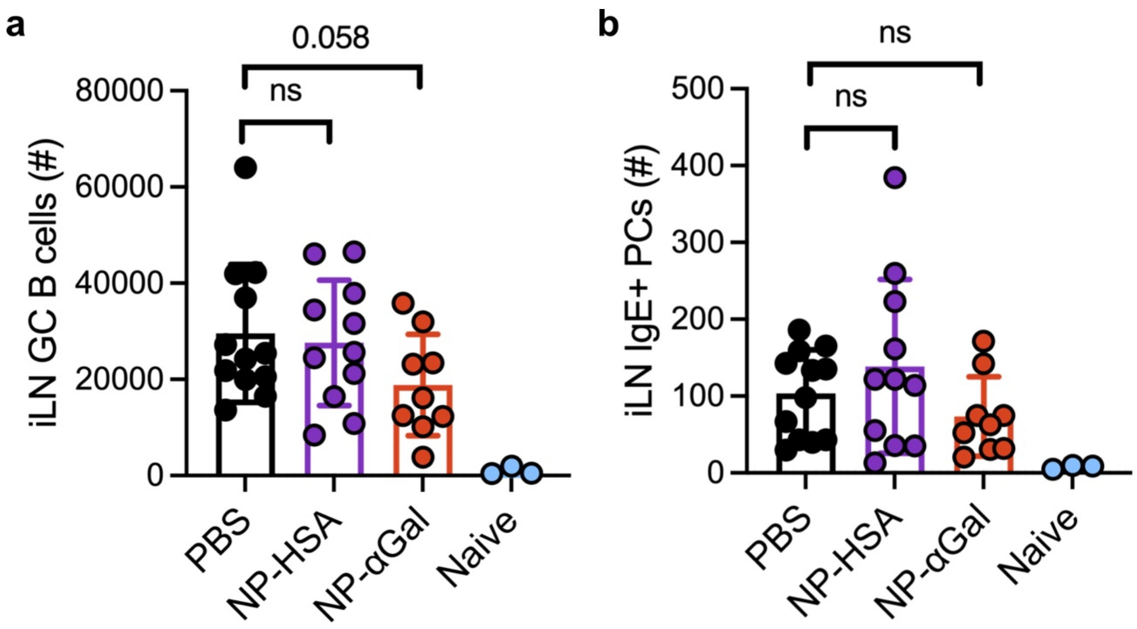

Supplementary Figure 1. Prophylactic administration of NP-αGal does not affect the numbers of germinal center B cells and IgE+ plasma cells. **a**, Total numbers of germinal center (GC) B cells and **b**, IgE+ plasma cells (PCs) in the skin-draining inguinal lymph nodes of mice given two doses of NPs or PBS, followed by three injections of tick extract, and analyzed at day 35. All data are expressed as mean ± SEM, with unpaired, two-tailed *t*-test.

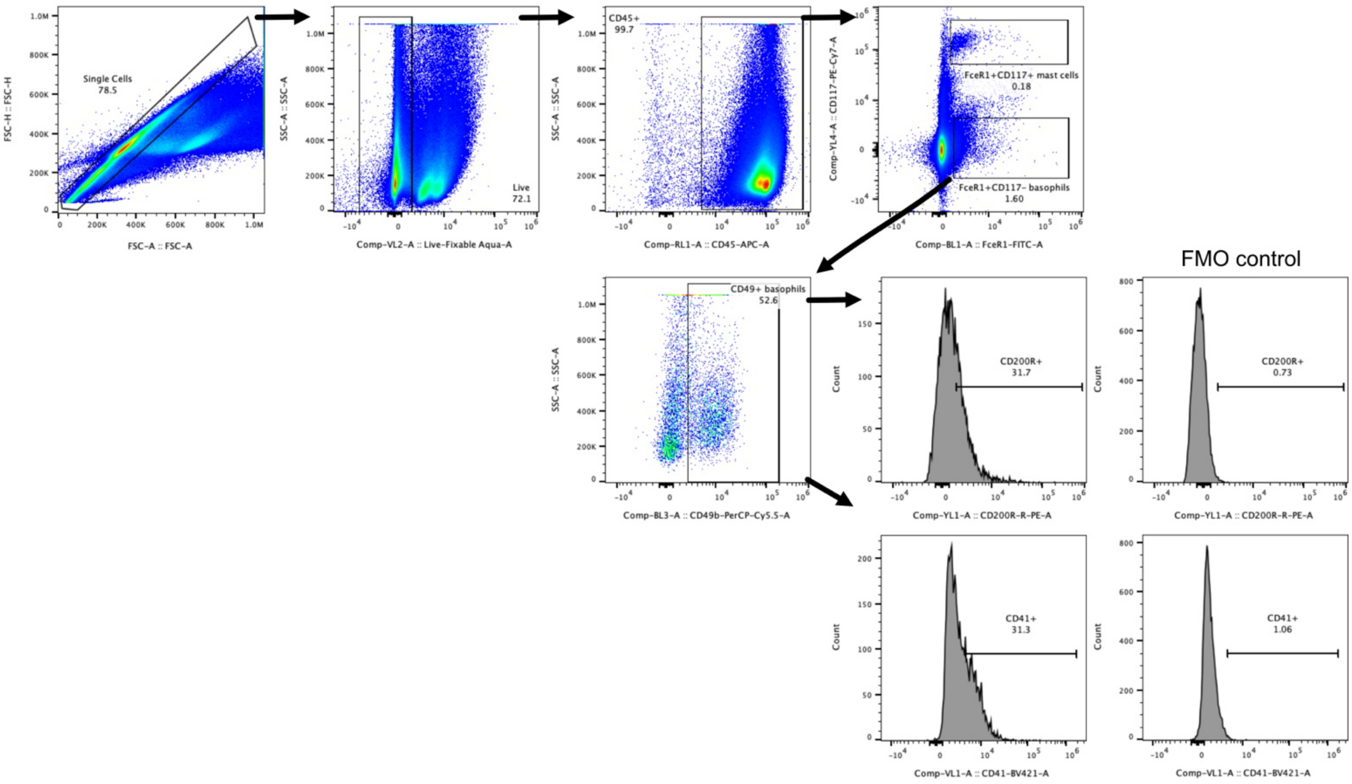

Supplementary Figure 2. Manual gating strategy for analyzing basophils and mast cells using flow cytometry. Gates were set for single cells, live, CD45^{+}, and then separated into FcεR1^{+}CD117 (c-kit)$^{+}$ to identify mast cells and FcεR1^{+}CD117^{-} cells. FcεR1^{+}CD117^{-} cells were further gated on CD49b to identify basophils and the expression of activation markers CD200R and CD41, with gates set based on fluorescence-minus-one (FMO) controls.

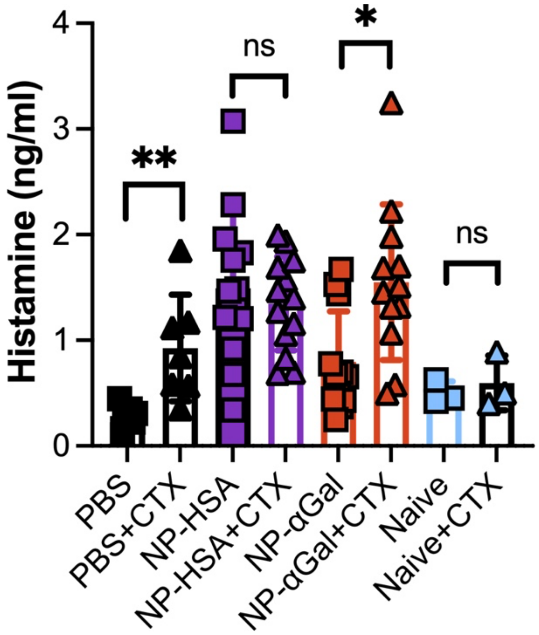

Supplementary Figure 3. Basophil activation test (BAT) for release of histamine after αGal stimulation with cetuximab. Heparinized blood from mice was incubated with and without cetuximab, and basophil release of histamine was measured by ELISA. All data are expressed as mean ± SEM. *P* = *0.05, with paired, two-tailed *t*-test.
